# On Emphysema of the Lungs as a Cause of Asthma

**Published:** 1830-01-01

**Authors:** 


XXVI.

### On Emphysema of the Lungs as a Cause of Asthma. By B. H. Coates, M.D., One of the Physicians to the Pennsylvania Hospital.*

The better informed members of the profession are now well aware of the abuses of the term asthma, as expressing an idiopathic disease. We venture to say that no more bulky tomes will be written on this subject, for, thanks to the cultivation of morbid anatomy, it is now ascertained that nine out of ten asthmatic cases are in point of fact instances of organic disease of the heart, great vessels, or lungs. We do not deny the existence of pure spasmodic asthma, but we do most boldly

* North Amer. Journ. July, 1829.

deny its frequency, and assert without fear of contradiction that by far the greater number of patients who are treated for this disease, are in reality suffering from organic disease. Of all the affections the ignorance of whose real nature is plaistered over on the part of the practitioner by this nosological salve, dilatations and aneurisms of the arch of the aorta are perhaps the most common. We have an instance of this under our notice at the present moment, in the person of an elderly man who has obviously dilatation of the arch of the aorta or innominata, and who was doctored and drugged for asthma. Dr. Coates has written a short paper in our American contemporary with the object of proving that emphysema of the lungs " is an abundant cause, or rather means of increasing and perpetuating" the disease, and we think the two cases which he brings forward are worthy of notice.

*Case* 1. " The notes of the following case were not prepared at the time. The dates are taken from records; while the description of the symptoms and of the morbid appearances, is drawn up from memory and inquiries. The statement of the results of dissection was subjected to the review and approbation of Dr. W. D. Gallaher, who kindly assisted me in the examination.

" John Golder, a seaman, a native of New Jersey, aged about 35 years, was admitted into the Pennsylvania Hospital, August 18, 1828. He complained of the symptoms usually given as characterizing spasmodic asthma. His thorax was found of a large size, equal on the two sides and rounded. The motion of inspiration was quicker than that of expiration. On tapping the thorax with the fingers, and, about the same time, doing so to a person with healthy lungs, placed alongside of him, the thorax of Golder emitted a sound by far more distinct and hollow. The difference was perfectly perceptible to a number of auditors assembled together. No such difference was found between the opposite sides of the thorax. The respiratory murmur was much less distinct than in the natural state. No striking rattle of any character was perceived.

" The patient was pronounced to suffer under emphysema of the lungs. He was placed under the common palliative treatment; consisting chiefly of strong anodynes, of occasional bleedings, and light diet of easy digestion. Temporary relief was experienced; and, after several fluctuations, he took his discharge on the 30th of August; and in the night of the 28th or 29th of September, he sent for me to his lodgings. I found him in a violent paroxysm, of which he had frequent returns, rapidly increasing in severity and continuity, until the 2d of October, when he died. These attacks were characterized by a dark and ghastly countenance, cold sweats, a pulse at last nearly imperceptible, incapability in general of resting in an inclined posture, and an anxious desire, even when excessively cold and unable to speak, of sitting by the open window. No other treatment remained to be tried than that employed before; and this was put in practice with exactly the same momentary relief. The patient had frequently complained of great distress in the central parts of his thorax; but *without any acute pain, tearing sensation, or other symptom particularly referrible to the arch of the aorta.*

" *Dissection.* On stripping the corpse, a slight projection of the upper bone of the sternum was observed. This had not been noticed during life. The body had lost much of its muscularity, but the cellular membrane was bulky.

" On opening the thorax, the lungs were found completely to fill the cavity, and did not collapse. The air cells were visible on the surface, and appeared about the size of millet seeds. Numerous small collections of air were also found in the cellular tissue of the lungs; as was evinced by the facility with which it could be impelled from one point to another with the finger. A small portion of serum was found in each pleura; which membranes were otherwise healthy. An unusual amount was also found in the pericardium; but this cavity and the heart were healthy.

"Behind the first bone of the sternum was found a large tumour of considerable solidity; which proved to be an aneurism of the arch of the aorta, embracing the origins of the arteria innominata, left carotid, and left subclavian. The sac was nearly as large as a man's fist; and had evidently been the cause of the projection of the first bone of the sternum.

"The trachea was found permanently changed in its figure where it had passed across the tumour. Its lining membrane was considerably inflamed; as was evinced by a smart red colour, and an increased amount of mucus.

"The abdomen presented nothing remarkable, and the head was not examined."

Dr. Coates believes that the aneurism was the original cause of the cough, the violence of which produced in its turn emphysema of the lungs and the asthmatic symptoms. Now we should be inclined to go even further than Dr. Coates, and attribute the spasmodic symptoms themselves, or in other words the asthma, to the tumour. Whoever has opened the bodies of many persons dead of diseases of the heart or great vessels, must have noticed that a more or less emphysematous state of the lungs is not unfrequent, independently of any well-marked asthmatic symptoms during life. We are of opinion that emphysema of the lungs may be produced in such cases during the last days of the patient's existence, when the orthopnœa and dyspnœa are almost always distressing, the blood ill oxygenized, and the struggle of the respiratory system great. Who is not aware that during the short time that intervenes between the invasion of apoplexy and its fatal termination, the lungs will most commonly become œdematous and emphysematous, loaded with serum and air?

*Case* 2. "W——H——L, who died aged two years and five days, was the child of healthy parents, natives of Denmark, and appeared to possess tolerable health, till about ten months old. About this time, being towards the end of winter, she appeared to suffer from repeated colds. To these, however, the parents did not think the assistance of a physician necessary. No peculiarity was then noticed in her cough. Early in the summer of 1828, she became affected with cholera infantum; to prescribe for which, my friend, Dr. A. B. TUCKER was sent for. Dr. T. treated the attack chiefly by astringents; and, after some teeth were cut, the child improved so much in health and flesh, that Dr. T. discontinued his visits. At that time the cough was not considered as of very particular consequence; and, about October, at eighteen months old, the child was weaned, in consequence of a deficiency in the supply of its parent's milk.

"About six weeks before its death, the child's cough became very severe, with great apparent danger of suffocation. It was found necessary to hold it upright during the paroxysms; which were convulsive and fatiguing. About the same time, it became subject to smart bleedings at the nose. It was observed to be worse after eating. An increased quantity of mucus was expectorated. It was partially relieved by syrup of squills, the only medicine which was given to it. A short time previously to its death, it was affected with diarrhœa; but whether this had been of long continuance, we have not ascertained. The parents suspected worms; as the child was observed to pick its nose. None, however, were discovered in its discharges. No symptoms were observed in any way indicative of pleuritis. Dr. T. was again called in, but found it in a dying state. The termination of its life took place soon after, apparently by actual suffocation.

"*Dissection*, April —, 1829, sixteen hours after death. Body not much emaciated; remarkably enlarged along the lower end of the thorax. A small quantity of yellowish serum ran from the nose.

"*Thorax*. *Right pleura* contains about an ounce and a half of yellowish serum. Its surface, vascular, and redder than that of the opposite side. An adhesion of considerable firmness, an inch and a half long, and three quarters of an inch wide, between the anterior part of the right lung and the mediastinum. Several large collections of *air* over various parts of the surface of both *lungs ;* particularly a large one in the cellular substance of the before-mentioned adhesion, and extending in front of the pericardium. These collections principally affected the seams or lines of junction of the different lobules of the lungs. Their inner surfaces were smooth, intersected at irregular distances, with imperfect partitions, and, in places, produced into long, polished tubes. Numerous thin whitish collections of tuberculous matter, arranged in granular masses, over the surface of the right lung. Lungs exhibit an unusual quantity of bronchial mucus when cut.

" *Left pleura* contains a little yellowish serum. A few of the same tuberculous infiltrations. Lungs otherwise similar.

" *Pericardium.* About half an ounce of yellowish, flaky serum in the cavity. Otherwise natural.

" *Thymus gland* and some adjacent *lymphatic glands,* hardened, nearly as hard as cartilage in some places. Whitish matter in some of the lymphatic glands. It appeared that the trachea, about its bifurcation, was pressed upon by the tumour.

" *Trachea.* Lining membrane, of a high, vascular redness.

" *Abdomen. Cavity* contains about an ounce of greenish serum.

" *Liver.* Not thought to be larger than belongs to the age of the subject (two years). Natural. Gall bladder full.

" *Stomach* natural. (Carefully examined.)

*Intestines. Small intestines* examined in various places. No unnatural appearance detected, but a very considerable quantity of bile.

" *Large intestines* extremely contracted. Sides in contact, except the cœcum, which was of its natural size Mucous membrane not at all reddened or ulcerated. Bile copious. Examined in the same way as the small intestines.

" *Head* not examined."

Neither is this in our opinion an unexceptionable example either of asthma or of emphysema of the lungs. Mere violent cough in paroxysms is not surely the only characteristic of asthma, but it is the only one discoverable in the foregoing case. Such symptoms as these appear to us to be easily accounted for by the pleurisy and tubercular infiltration in the substance of the lungs. The partial extravasations of air were probably the consequences rather than the cause of the cough. The cases, however, are interesting in a pathological point of view, and we hope their perusal will stimulate our readers to prosecute zealously the examination into the many organic causes of asthmatic complaints. We assure them that the results will repay their trouble.

---

